# Adoption of High-sensitivity Troponin Testing and Emergency Physician Ordering Behavior

**DOI:** 10.5811/westjem.2022.2.54242

**Published:** 2022-04-04

**Authors:** Nicole R. Hodgson, Katie L. Kunze, Elisabeth S. Lim, Steven A. Maher, Stephen J. Traub

**Affiliations:** * Mayo Clinic Arizona, Department of Emergency Medicine, Phoenix, Arizona; † Mayo Clinic Arizona, Department of Quantitative Health Sciences, Phoenix, Arizona; ‡ Brown Alpert School of Medicine, Department of Emergency Medicine, Providence, Rhode Island

## Abstract

**Introduction:**

Emergency departments (ED) are rapidly replacing conventional troponin assays with high-sensitivity troponin tests. We sought to evaluate emergency physician utilization of troponin tests before and after high-sensitivity troponin introduction in our ED.

**Methods:**

We retrospectively examined 9,477 ED encounters, identifying the percentage in which physicians ordered a serum troponin both before and after our institution adopted a high-sensitivity troponin test.

**Results:**

After introduction of high-sensitivity troponin testing, the percentage of ED encounters in which physicians ordered troponin studies decreased (28.3% before vs 22% after; P <.001), with the drop most pronounced in admitted patients (decrease of 10.9% [95% confidence interval [CI]: 7.3%–14.5%] in admitted patients vs decrease of 3.6% [95% CI: 1.7%–5.4%] in discharged patients; P<.001)

**Conclusion:**

Introduction of high-sensitivity troponin testing was associated with a decrease in troponin ordering. While the reasons for this are unclear, it is possible that physicians became more selective in their ordering behavior because of the lower specificity of high-sensitivity troponin.

## INTRODUCTION

Introducing high-sensitivity cardiac troponin (hs-cTn) in emergency departments (ED) often improves ED length of stay^1^,^2^ and can lead to lower stress-test utilization.^1^ However, age, renal dysfunction, hypertension, peripheral artery disease, prior myocardial infarction, and use of diuretics are associated with elevated hs-cTn outside of acute coronary syndrome (ACS).^3^,^4^ Decreased specificity of hs-cTn compared to conventional assays along with results in the indeterminate range for ACS may lead to emergency physician (EP) uncertainty, altering EP heuristic pathways. We sought to evaluate EP utilization of troponin tests before and after hs-cTn introduction in our ED.

## METHODS

We performed a retrospective analysis of ED operational data. Our institutional review board provided an exemption from full review.

The Mayo Clinic Arizona ED is a tertiary care facility serving approximately 34,000 patients yearly with 26 rooms and up to nine hallway spaces in Phoenix, AZ, staffed by residency-trained EPs. There is no fast track or ED observation unit. Our EPs acquire patients on a rotational assignment basis, with no practical discretion as to which patients they evaluate. As EPs receive patients to individual queues when patients arrive in triage, we employ no triage physicians. The EPs place triage orders on their assigned patients to expedite care prior to physical assessment. Due to this front-end workflow, we rarely use nursing-initiated order sets outside of protocol activations such as acute stroke or ST-elevation myocardial infarction. Residents rotate through the ED and see approximately 5% of patients. No nurse practitioners or physician assistants work in our department.

Our ED replaced conventional Roche fourth generation troponin T with Roche fifth generation hs-cTn on July 17, 2018. We reviewed all ED encounters seen by full-time EPs from July 17–September 16, 2018 (the “after” period). We chose this end date as it coincided with the rollout of a new electronic health record (EHR), which we believed would introduce additional confounders to our analysis. To account for seasonal variability, we matched this timeframe with a similar period one year prior, examining all ED encounters staffed by full-time EPs from July 17–September 16, 2017 (the “before” period). To limit the influence of different EP ordering practices,^5^ we excluded patients seen by part-time EPs (who work inconsistent clinical hours), EPs employed during only one of the assessment periods, and encounters missing an assigned physician.

We obtained general characteristics of patient encounters in both groups including age, gender, race, Emergency Severity Index (ESI), ED length of stay (LOS), and admission rates. We examined the percentage of encounters receiving ED orders for a troponin test, either conventional troponin T before or hs-cTn after. We determined this percentage for all ED patients and then split the data to separately examine discharged and admitted (including hospital observation) patients. To determine whether overall EP testing behavior changed, we performed this same analysis for hemoglobin, a common ED test. We selected hemoglobin since the data was readily available in our operational database.

We used descriptive statistics (counts, percentages, means, and standard deviations), chi-square tests, and Kruskal-Wallis test by ranks where appropriate to examine differences in demographics in the before and after groups, as well as rates of troponin and hemoglobin ordered before and after hs-cTn overall and by type of encounter (discharge vs admission). Confidence intervals (CI) for the differences in rates of testing between groups were constructed using the Chan-Zhang exact method for calculating CIs for differences of binomial proportions.^6^ All analyses were performed using SAS version 9.4 (SAS Institute Inc., Cary, NC, USA) and R via Rstudio (Boston, MA) and the *arsenal* package.

## RESULTS

Appendix 1 details excluded encounters. We report demographic characteristics of the before and after hs-cTn groups in Table 1.

We report counts and rates of ED encounters receiving a troponin test in the before and after hs-cTn groups in Table 2. Encounters with an order for a troponin test decreased after introduction of hs-cTn (28.3% before vs 22% after; P<.001), with the drop appearing most pronounced in admitted patients (decrease of 10.9%, 95% CI: 7.3–14.5% in admitted vs 3.6%, 95% CI: 1.7–5.4% in discharged patients; P<.001).

Unlike troponin ordering, hemoglobin ordering did not change after hs-cTn introduction (70.2% before vs 69.5% after; P = .48). Hemoglobin ordering behavior remained the same when examining subcategories of discharged (56.8% before versus 56.8% after, P = .98) and admitted (98.1% before versus 98.0% after, P = .83) encounters.

## DISCUSSION

Our results demonstrate a significant decrease in ED encounters receiving a troponin test after introduction of hs-cTn. We believe our EPs became more selective in their troponin ordering behavior. One study examining conversion to hs-cTn’s impact on laboratory workload noted a decrease in troponin tests after conversion to hs-cTn, with a decline in test orders of over 10% despite an increase in total ED visits, with an overall decrease in percentage of ED encounters receiving a troponin study.^7^ Although the authors did not speculate as to the cause, we suspect EPs consciously or unconsciously adjusted their ordering behavior to accommodate the decreased specificity of hs-cTn in their diagnostic heuristics. This is supported by informal discussions with several of our physicians, who expressed frustration when having to navigate indeterminate hs-cTn results.

Decisions made by EPs change throughout a shift, with EPs picking up fewer patients and making more decisions that shorten ED LOS near end of shift (EOS).^8^ As our EPs are automatically assigned patents, they do not have the ability to cherry-pick easier patients or take fewer patients near EOS. Anticipation of an indeterminate troponin requiring repeat for trending near EOS may lead EPs to be more discriminant in their hs-cTn orders. Emergency department managers employing patient assignment models should be aware of this possibility when incorporating hs-cTn and make operational adjustments to ensure that all patients continue to receive high quality care.

Increased ED troponin-ordering selectivity may be harmful or beneficial to patients. Although classic teaching recommends maintaining high suspicion for ACS, especially in patient groups who present atypically, some evidence suggests that EPs may overtest.^9^ One study of ED patients over age 65 presenting with nonspecific complaints (such as generalized weakness, fatigue and dizziness) found that although 20% of these patients had positive troponins, 93.8% of these elevations were due to factors other than ACS (most frequently sepsis) and none of the patients received reperfusion therapy.^10^ After the institution of hs-cTN, physicians at our ED may have deferred troponin evaluations of these patients, either using gestalt alone or in conjunction with an electrocardiogram (ECG).

If so, this approach may have pitfalls. Although the above method may be safe in geriatric patients with nonspecific complaints, ED patients presenting with chest pain are another matter. In a separate study, physician gestalt, even combined with an ECG, did not identify all ACS cases.^11^ Reducing testing in this group of patients may prove dangerous.

## LIMITATIONS

Our study suffers from several limitations. The first is the capability of our EHR: the free-text nature of chief complaints at the time of data collection limited our ability to determine the percentage of ED visits with chest pain and potential ACS equivalents. Fewer patients with chief complaints suspected to be ACS equivalents may have presented to our ED in the after period. However, Table 1 suggests that our patient population remained similar on most demographic and other patient characteristics such as admission rate, suggesting similar severity of illness in the before and after periods.

Patients in the after group were more likely to be labeled ESI 1 and 2, which we believe would typically prompt more orders for troponins, the opposite of our observed trend. Hemoglobin ordering, unlike troponin ordering, did not decrease after introduction of hs-cTn, suggesting that overall resource utilization remained similar before and after hs-cTn. We believe that matching the time of year when selecting the comparison period mitigated any effect of seasonal variability of complaints, and excluding encounters seen by part-time physicians and physicians employed during only one time period mitigated individual physician-ordering variability. We performed no specific interventions or community outreach programs during this time which would have changed the nature of presenting complaints.

A second limitation is that ours is a single center, retrospective review; thus, we can comment on correlation but not causation. However, our findings do align with other studies noting a decrease in percentage of ED encounters receiving a troponin study after introduction of hs-cTn.^7^ Due to the change in our EHR, we only examined a two-month period post hs-cTn; the decrease in ordering behavior may represent a period of acclimation and not long-term behavior. We hope our study prompts additional investigation into whether these findings persist.

## CONCLUSION

After introduction of hs-cTn, the percentage of ED patients receiving troponin studies decreased. We suspect that emergency physicians became more selective in their ordering behavior to account for the lower specificity of hs-cTn.

## Supplementary Information





## Figures and Tables

**Table 1:** Encounter characteristics before and after hs-cTn introduction.

Characteristic	Before hs-cTn	After hs-cTn	P-value
Gender			
Female (%)	2,774 (53.7%)	2,298 (53.3%)	0.703^1^
Mean age in years (SD)	57.5 (20.9)	56.4 (21.0)	0.020^2^
Race			
White (%)	4,600 (89.7%)	3,799 (89.3%)	0.548^1^
ESI (%)			0.017^1^
1	59 (1.1%)	51 (1.2%)	
2	1,405 (27.3%)	1,274 (29.6%)	
3	3,116 (60.5%)	2,512 (58.4%)	
4	520 (10.1%)	440 (10.2%)	
5	50 (1.0%)	23 (0.5%)	
Missing	16	11	
ED length of stay in minutes (SD)	227.2 (161.2)	225.3 (136.8)	0.238^2^
Inpatient length of stay in hours (SD)	80.3 (102.0)	85.3 (99.9)	0.071^2^
Admission Status			
Discharged (%)	3,491 (67.6%)	2,982 (69.2%)	0.097^1^

*hs-cTn*, high-sensitivity cardiac troponin; *SD*, standard deviation; *ESI*, Emergency Severity Index; *ED*, emergency department.

1 chi-square p-value;

2 Kruskal-Wallis P-value.

**Table 2:** Number (%) of ED encounters with troponin ordered before and after hs-cTn introduction.

Encounter type	Before hs-cTn	After hs-cTn	*P*-value*
All encounters	1,463 (28.3%)	948 (22.0%)	<.001
Discharged	657 (18.8%)	454 (15.2%)	<.001
Admitted	806 (48.1%)	494 (37.2%)	<.001

*hs-cTn*, high-sensitivity cardiac troponin.

* *P*-values from chi-square analyses.
