# Supplementary material for: Adoption of High-sensitivity Troponin Testing and Emergency Physician Ordering Behavior
Source: West J Emerg Med. 2022 Apr 4;23(3):439–42. doi: 10.5811/westjem.2022.2.54242 (PMC9183784; doi:10.5811/westjem.2022.2.54242)
Supplement: Supplementary file 2 [file wjem-23-439-s002.docx]

APPENDIX 1. Included and excluded encounters.

There were 11,307 encounters during the study period: 5591 during the before period and 5716 during the after period. We excluded 1830 records (425 from before and 1405 from after), leaving 9477 records for analysis. Before- period exclusions included 35 encounters missing an assigned physician and 390 encounters seen by a part-time emergency physician (EP). After-period exclusions included 47 encounters missing an assigned physician, 802 encounters seen by a part-time EP, and 556 encounters seen by new hires not employed during the before period.
